# Fostering regulatory processes using computational scaffolding

**DOI:** 10.1007/s11412-023-09388-y

**Published:** 2023-03-27

**Authors:** Leonardo Silva, António Mendes, Anabela Gomes, Gabriel Fortes

**Affiliations:** 1 University of Coimbra, CISUC, DEI, Rua Sílvio Lima, Polo II, Coimbra, Portugal; 2 Federal Institute of Technology in Pernambuco, Rua Padre Agobar Valença, Garanhuns, Pernambuco Brazil; 3 Coimbra Institute of Engineering (ISEC), Rua Pedro Nunes, Coimbra, Portugal; 4 Universidad Alberto Hurtado, Almte. Barroso 10, Santiago, Chile

**Keywords:** CSCL, Socially shared regulation of learning, Coregulation, Self-regulation of learning, Programming learning

## Abstract

The use of computational scaffolding is a crucial strategy to foster students’ regulation of learning skills, which is associated with increased learning achievement. However, most interventions treat the regulatory processes as individual actions isolated from a social context. This view contradicts the most recent research that points to the importance of studying the regulatory phenomenon from a social-cognitive perspective, where students’ interactions influence their regulation of the learning process. This work explores these problems and presents multiple scaffolds to promote Self-regulation of Learning (SRL), co-regulation, and socially shared regulation of learning (SSRL) embedded within a computer-supported collaborative learning environment. A single-blind randomized controlled trial was performed with students (*n* = 71) enrolled in an online introductory programming course. Students were randomly assigned to three groups: 1) SRL-only support, 2) SRL, co-regulation, and SSRL support, and 3) a no support control group. The findings revealed that students who received regulatory support achieved higher course grades than the control group. However, only students who received SSRL and co-regulation support achieved superior performance in collaborative activities, confirming the importance of this type of regulation. Even though students did not increase in SRL aptitude, the intervention provided support for achieving higher grades in the course.

## Introduction

Students’ control of their mental resources to achieve educational goals is commonly referred to as self-regulation of learning (SRL). It is an ability that involves cognitive, metacognitive, motivational, emotional, and behavioral processes, which together support the planning, execution, and self-assessment of academic tasks (Zimmerman & Schunk, 2011).

SRL is necessary for successful learning in multiple educational contexts, including programming education (Loksa et al., 2022). However, students often face regulatory difficulties that can negatively impact their academic performance (Loksa et al., 2020). With appropriate support, it is possible to enhance the use and development of regulatory skills (Theobald, 2021). One example is the use of computer-based learning environments (CBLE) that incorporate regulatory scaffoldings, as these have been shown to significantly improve students' academic performance (Zheng, 2016a).

In the context of introductory programming education, several CBLEs were proposed to support SRL (Loksa et al., 2022). However, most of them conceptualized the regulation of learning as an individual process, neglecting the potential influence of the social context (Silva et al., 2021a). This view contradicts recent research that points to the importance of social interaction as a key component of the regulatory process and an enabler of collective goal attainment (Malmberg et al., 2017). These aspects are theorized under the psychological constructs of Socially Shared Regulation of Learning (SSRL) and co-regulation (Panadero et al., 2015).

SSRL refers to the regulation of learning episodes within a group of people who collectively negotiate and deliberate regulatory strategies to achieve a common shared goal (Hadwin et al., 2017). Co-regulation refers to regulatory stimulation that arises from interpersonal interactions. Thus, one’s regulatory processes are temporally guided by another, resulting in SRL occurrence. Co-regulation can also occur in groups and promote SSRL episodes.

SRL is associated with an established body of literature cataloging stimuli that can mediate this process, while the understanding of how to design social regulation scaffoldings (namely co-regulation and SSRL) needs further investigation (Järvelä et al., 2019). Moreover, there is also an opportunity to investigate how individual and social regulation scaffolding can be combined. This work aims to contribute to the aforementioned scientific knowledge by presenting the design and evaluation of a Computer-Supported Collaborative Learning (CSCL) system named Regulatory Support for Programming Education (RESPE). The objective is to explore the use of computational scaffolding to promote individual and social regulation of learning. Although the educational context of this work is STEM education, the proposed pedagogical design might be applied to other areas in future work accompanied by appropriate adaptation.

The proposed software was evaluated through a randomized, single-blind controlled experiment over ten weeks with 72 students enrolled in online introductory programming courses. The objective was to assess its effectiveness in supporting learning of programming and the development of regulation of learning skills. Two research questions guided this investigation: RQ1) Do students who received regulatory support achieve higher performance in learning of programming as compared to students who did not receive that support? and RQ2) Do students who received regulatory support develop higher regulatory skills as compared to students who did not receive support?

The main contributions of this study are: i) a pedagogical design for supporting social regulation and SRL in introductory programming education; ii) statistical evidence related to its effectiveness for supporting learning of programming through scaffolding related to regulation of learning; and iii) a description of the pedagogical design process and the challenges encountered, which is meant to serve as a reference for the design of future CSCL environments.

### Regulation of learning

Regulation of learning is the ability to become aware of and manage one’s own mental functioning in order to achieve academic goals (Schunk, 2005). This construct encompasses students’ ability to use their mental resources to execute educational tasks, such as 1) cognition to create knowledge of the task and define the strategies needed to perform it; 2) metacognition to manage how one’s mental faculties operate, allocating or revising these resources throughout a task; 3) behavior to control one’s own conduct towards improving the ability to perform a task, and finally, 4) emotion and motivation to manage one’s own goal-orientation and affective state in order to perform the task effectively (Zimmerman & Schunk, 2011).

Multiple models have been developed to describe the regulation of learning processes and to help understand how learning occurs, as well as the strategies used by students for managing that process. The model developed by Barry Zimmerman is one of the most used (Panadero, 2017), including in programming education research (Silva et al., 2021a). This model describes SRL as a cyclical process comprising three phases. During the first phase, which is named *forethought*, students employ cognitive strategies to understand and plan the learning task, with the emergence of motivational beliefs that drive their interest in engaging in it. The second phase is named *performance* and describes a set of actions occurring during the execution of the learning activity, such as those of a metacognitive nature (e.g., monitoring), cognitive strategies (e.g., drawing a flowchart to predict the behavior of a computer program), and also motivational (e.g., evaluating consequences). Finally, the last phase is *self-reflection*, where students are expected to make judgments about their performance (whether positive or negative) and reflect on how they can improve. Thus, the acquired knowledge feeds into the next cycle of SRL. This model was used as a reference in the design of our proposed regulatory scaffolding.

A characteristic of SRL research that has been the subject of some critique is the conceptualization of the student as an individual, which concise his/her learning in isolation from the social environment and the classroom’s collective activities (Hadwin & Oshige, 2011; Zimmerman, 1990). In stark contrast, according to multiple other educational theories, learning is inherently a social process, and thus the relationships established among students are expected to influence their regulatory processes (Panadero & Järvelä, 2015; Schunk, 2012).

This work adopts a socio-cognitive perspective of regulation of learning, recognizing that regulation can occur individually (SRL) and also through social interactions, as characterized under the theoretical frameworks of Socially Shared Regulation of Learning (SSRL) and co-regulation (Hadwin et al., 2017). SSRL emerges in group interactions, as participants actively collaborate to achieve a shared learning objective (Hadwin & Oshige, 2011). In this sense, students jointly employ multiple regulatory actions, defining strategies for solving a task, creating awareness of their peers’ concerns, and aggregating the multiple perceptions of group participants (Järvelä et al., 2014). Meanwhile, co-regulation involves regulatory stimuli that an individual receives from another subject, such as a person or a software agent. In this way, an individual's regulatory processes are guided in process towards successful SRL by triggering SSRL episodes when they are needed in groups over time.

Within the scope of this work, we did not differentiate co-regulation from SSRL. Thus, considering that both of these characterizations of regulation of learning emerge from social interactions, we opted to fold them together under the term social regulation (Hadwin et al., 2017; Järvelä et al., 2014).

#### Fostering regulatory processes using computational scaffolding

According to the literature, self-regulation can be improved with proper guidance, such as using computational scaffolds (Duckworth et al., 2009). This type of intervention has many benefits, such as the possibility of scaling the support capacity, collect and process data in real-time to enable diagnosis of regulatory capabilities, and provide tailored support that is optimal for each student (Järvelä et al., 2014).

Several strategies might be used as the basis of computational scaffolding meant to foster the regulation of learning. Some of these scaffolds, such as learning diaries, were originally designed for non-technological environments but have been adapted for use in a digital contexts (Schwendimann et al., 2018). Other strategies, such as learning analytics, only exist due to the resources provided by computers, enabling the collection of data about students' learning processes (Law et al., 2017).

Strategy selection depends on which psychological processes are intended to be stimulated. For example, sending notification emails to remind students about incomplete tasks could be used to support their learning process monitoring, whereas asking students to set their learning goals is an important approach for motivational regulation (Schunk, 1990).

#### Mediating social regulation using CSCL environments

CSCL environments are a form of educational software designed to provide opportunities for students to interact while experiencing stimulation of their cognitive, motivational, metacognitive, and social processes (Jeong & Hartley, 2018). Evidence points to the effectiveness of this software in multiple educational contexts, but also points to enduring challenges in effectively supporting productive collaboration (Jeong et al., 2019).

Research has shown that social regulation is a central element of learning in collaborative activities (Järvelä & Hadwin, 2013). At the same time, without proper regulatory support, students would have difficulty managing their shared goals and achieving productive collaboration (Hadwin et al., 2017). Therefore, a significant part of CSCL research has explored how social regulation could be stimulated within these environments (Zheng, 2016b). The objective is to provide opportunities for students to collaboratively plan, monitor, and evaluate their work (Panadero & Järvelä, 2015).

Järvelä et al. (2014) proposed a series of design principles for social regulation stimuli. It is necessary to consider how group members can externalize and raise awareness about their learning processes and that of others. In this sense, the CSCL environment is vital for providing resources to activate the regulatory processes and foster their externalization among group members.

Some concrete examples of the aforementioned theoretical propositions are the Radar, OurPlanner, and OurEvaluator (Järvelä & Hadwin, 2013). These resources used questions and graphs to prompt and activate regulatory processes related to the planning and monitoring of collaborative activities. However, despite the advances in the area, there is still a need to explore other forms of scaffolding and provide evidence regarding their effectiveness (Lin, 2018; Panadero & Järvelä, 2015).

### Regulation of learning research in programming education

The use of SRL theory to study learning of computer programmng has gained traction in recent years (Loksa et al., 2022). Nevertheless, many questions already consolidated in other educational contexts are still open, such as how SRL occurs in this learning domain and how it could be stimulated (Prather et al., 2020). In addition, there is still an opportunity for researchers to understand how SRL theory aligns with the distinctive aspects of computer programming instruction (Silva et al., 2021b).

Initial findings support an association between SRL and achievement in the domain of computer programming (Loksa et al., 2022). Researchers were interested in identifying the regulatory strategies that emerged in this learning domain, which were classified as general-purpose regulatory strategies, such as the establishment of educational goals and metacognitive monitoring, and programming specific regulatory strategies, such as drawing flowcharts^1^ (Falkner et al., 2014).

Falkner et al. (2015) found that novice programming students rely on general regulatory strategies and gradually show increased usage of programming specific regulatory strategies. This characteristic points to the relevance of studying the SRL processes according to the specificities of the programming education field. At the same time, the author points to the need to stimulate those regulatory skills that beginners do not usually possess.

#### Computer-based learning environments grounded on principles from the theory of regulation of learning in programming education

Multiple CBLEs have been designed to foster cognitive, metacognitive, motivational, and emotional processes during computer programming instructional activities (Prather et al., 2020). Thus, it is assumed that this scaffolding will support students’ regulatory processes and enhance their learning of computer programming (Loksa et al., 2022).

In a previous systematic literature review, we identified several regulatory scaffolds used in existing CBLEs for the programming education domain (Silva et al., 2021a). Most of this work used scaffolds adapted from other educational contexts. For example, Alhazbi (2014) requested students to write a learning journal about their studying plans, evaluate their progress, and reflect on their learning processes, all of which are SRL abilities. This scaffolding exemplifies a class of general-purpose stimuli that can be used regardless of the educational area. One significant finding from our survey was that only a few CBLEs in the literature have used scaffolding to support the software development process, which makes this point in time a critical moment for the learning of computer programming. Finally, most studies have focused on supporting SRL, disregarding the social context’s influence on the regulatory process (Garcia et al., 2018; Loksa et al., 2022). This problem is not unique to computer science education, as other researchers have pointed out that the lack of studies dedicated to fostering social regulation in CSCL impacts the understanding of how to design these stimuli effectively (Panadero et al., 2015).

It is clear that for regulatory support to be effective, it needs to be stimulated in both individual (SRL) and social regulation dimensions (Hadwin et al., 2017). The present study was inspired by this integration of prior research and aims to present the design and evaluation of computational scaffolding that combines SRL and social regulation support considering the specific characteristics of computer programming education.

#### Computer-supported collaborative learning in programming education

Social interactions play a fundamental role in supporting students’ learning related achievement in programming education (Umapathy & Ritzhaupt, 2017). Thus, CSCL research has recently gained significant attention in the programming education field, aiming to take advantage of shared knowledge building.

In a previous literature review, we identified several characteristics of CSCL environment research situated in programming education (Silva et al., 2020). Most of them have focused on the cognitive aspects of the learning process, providing resources for students to collectively solve programming activities. One such example is using shared code editors for the joint realization of the problem-solving and program creation processes. At the same time, the findings point out that most published studies have not address elements related to social regulation, despite the importance of these processes for successful collaboration (Järvelä & Hadwin, 2013). Two important exceptions are the work presented by Li et al. (2022) and Gogoulou et al. (2012).

Li et al. (2022) used a shared documents platform to support collaborative computational thinking learning. Several activities were used to facilitate SSRL, such as asking students to discuss ideas for solving the task, assessing group progress, and evaluating one’s own accomplishments. The findings point to the effectiveness of providing this support to increase computational thinking performance. Gogoulou et al. (2012) presents the software e-Eclip used to support collaborative programming activities. This environment guided students’ dialogues fostering productive collaboration and creating awareness among them, which are elements deemed crucial for regulation in social contexts (Hadwin et al., 2017). Students who used the environment presented higher programming performance and greater receptivity to work in group activities.

Even though the above work presents important scaffolding for encouraging social regulation, they are limited to certain types of collaborative activities and do not offer support during program development. Thus, we propose novel scaffolding in this work that seeks to overcome some of the highlighted limitations by investigating the regulatory phenomenon from both individual and collaborative perspectives.

Understanding how social regulation occurs within the programming education domain is still in its early stages. The findings reported by Prather et al. (2022) show that social strategies, such as help-seeking and group learning, are important tools students can use to overcome difficulties. In addition, the findings discuss how the regulatory strategies differ when students work individually or collectively. For example, goal setting has increased importance in self-regulation research. However, in collaborative activities, students value most opportunities to discuss coding tasks and request help. The authors conclude that the nature of collaborative programming activities aligns with Hadwin’s SSRL model (Hadwin & Oshige, 2011), showing that students experience multiple regulatory processes through their collective work.

### Regulatory Support for Programming Education (RESPE)

This study presents RESPE, a CSCL environment designed to support programming learning by assisting students in their self-regulated learning (SRL) and social regulation. The software includes several scaffolds aiming to foster students’ SRL and social regulation, covering the gamut of psychological areas, such as cognition, metacognition, behavior, and motivation. These scaffolds provided a basis for students to plan, monitor, and self-evaluate their performance during programming activities.

The software follows the Creative Commons Attribution-NonCommercial 4.0 International (CC BY-NC 4.0) license, and its source code is available at https://github.com/lsoaresesilva/32bits.

#### Pedagogical design

Designing educational software based on the regulation of learning constructs is a complex process (Azevedo & Hadwin, 2005). There are multiple views about how regulation occurs (Panadero, 2017) and the psychological areas present in this process each have their own nuances (Zimmerman & Schunk, 2011). In addition, it is necessary to take into account the specificities of the educational context where the software will be used (Dignath & Büttner, 2008). Lastly, programming education has its own set of unique considerations, such as the limited knowledge on how to foster social regulation of learning (Silva et al., 2021a).

To deal with this ill-defined scope, we opted for an incremental software design and development process, following the Design-Based Research methodology (DBR), which is well suited to these situations (Reimann, 2010). DBR divides the research work around cycles, each with its activities.

The following cycles were performed in this investigation: 1) create an understanding of the regulation of the learning process; 2) design and develop the RESPE learning platform; and 3) evaluate the RESPE learning platform. In the first cycle, two systematic literature reviews were conducted. The objective was to identify CBLEs grounded in the regulation of learning in the programming education domain and the scaffolds they have used.

The findings from the first review were used to build a conceptual framework describing multiple features to foster SRL in programming education (Silva et al., 2021a). However, a limitation of the framework was the absence of strategies to stimulate social regulation since the identified studies only focused on individual regulation. To address this gap, a second literature review was conducted with a broader scope to identify CSCL environments in programming education (Silva et al., 2021b). The findings from this review not only corroborated the lack of social regulatory support but also provided insights into how cooperative and collaborative activities could be explored in programming education.

Due to the dearth of studies exploring social regulation in programming education, we developed our own scaffolds based on design principles proposed by Järvelä et al. (2014): 1) make students aware of their processes, as well as those of their peers; 2) allow students to externalize their learning processes; 3) provide support to activate regulatory processes. These principles were adapted to the context of collaborative programming activities.

The first proposal of the pedagogical design underwent expert review by a cognitive psychologist who specialized in SRL research and two professors with more than 15 years of experience in programming education research. They validated the model’s adequacy for fostering learning regulation and support programming learning. During this process, the experts raised several questions: Does the student perceive the usefulness of the features? Will it be used during their learning? How will it be used? These were sought to be answered in the second cycle.

In the second cycle, a pilot study was performed to validate the questions presented in the previous paragraphs. The software prototype was developed and assessed over ten weeks during a pilot study. An online programming course was offered in January 2021 and was attended by ten students. They signed a consent form allowing their interaction with the software to be logged, such as mouse clicks to access specific features, chat logs, and their writing in the learning journal, among others. In addition, a questionnaire was administered anonymously, consisting of open-ended questions about students’ opinions on each of the proposed features, following the pattern: “How do you assess the usage of the < feature name > ”. A total of eight questions were used related to the SRL features presented in Table 1.

**Table 1:** Scaffolds used to support the regulation of the learning process in the RESPE platform

Scaffolding	Description	Focus
Learning journal	A journal where students plan and reflect on their learning processes	SRL
Software planner	A set of questions that guide students’ planning of their computer programming	SRL
SRL tips	Textual tips on the importance of SRL for learning	SRL
Intelligent agent	An intelligent agent that monitors students’ errors to present textual tips for improving their problem-solving behavior. In addition, suggestions for learning materials are presented	SRL
Error visualization	A pie chart containing the student’s main programming errors so that they can create awareness of their difficulties	SRL
Debug	An artifact that allows the visualization of the code execution flow and the variable values, which is helpful for identifying and correcting programming errors	SRL
Learning dashboard	A learning dashboard that presents several educational metrics that foster metacognitive awareness and support of self-reflection	SRL
Error analysis	An artifact that presents feedback on the main causes of errors, along with suggestions for how to correct the programming code	SRL
Collaborative software planner	A resource similar to the software planner but for use in collaborative activities	Social regulation
Ranking	A ranking that presents the five students with higher performance in the course. The feature was designed to foster students’ extrinsic motivation	Social regulation
Dialogue support	An intelligent agent that detects students’ interactions in the chat; in their absence, different messages are presented to improve communication within the group	Social regulation
Forum	A forum where students could interact with each other on themes related to their course	Social regulation
Help request	An opportunity for students to request help from their peers	Social regulation
Course progress	An artifact that presents the student’s course progress, consisting of a progress bar with the course conclusion and uses different colors to highlight the exercise’s conclusion status	SRL
Exercise solution	Affordance for students to visualize the exercise solution created by the teacher	SRL

The log analysis and responses to the questionnaire revealed some unexpected behaviors. For example, one student reported privacy issues, not knowing if their data would be shared with their peers. Students also stressed their difficulties in reflecting on the learning process, a problem also reported by Loksa et al. (2020). Some students complained about the repetitiveness or difficulty in using some scaffolds, and others did not see their usefulness.

The data collected in the pilot study corroborated the experts’ concerns about how students use the features of collaborative environments. Thus, in the third cycle, before the experiment was conducted, several modifications were introduced in RESPE. First, students were informed that their data would not be shared with their peers. Second, examples of how to use some of the scaffolds were included. Third, the learning journal was modified to present different questions every week instead of a fixed set. Finally, tips on the importance of SRL were included to stimulate students’ interest in self-regulating, which is critical for its realization (Theobald, 2021).

**Fig. 1:**
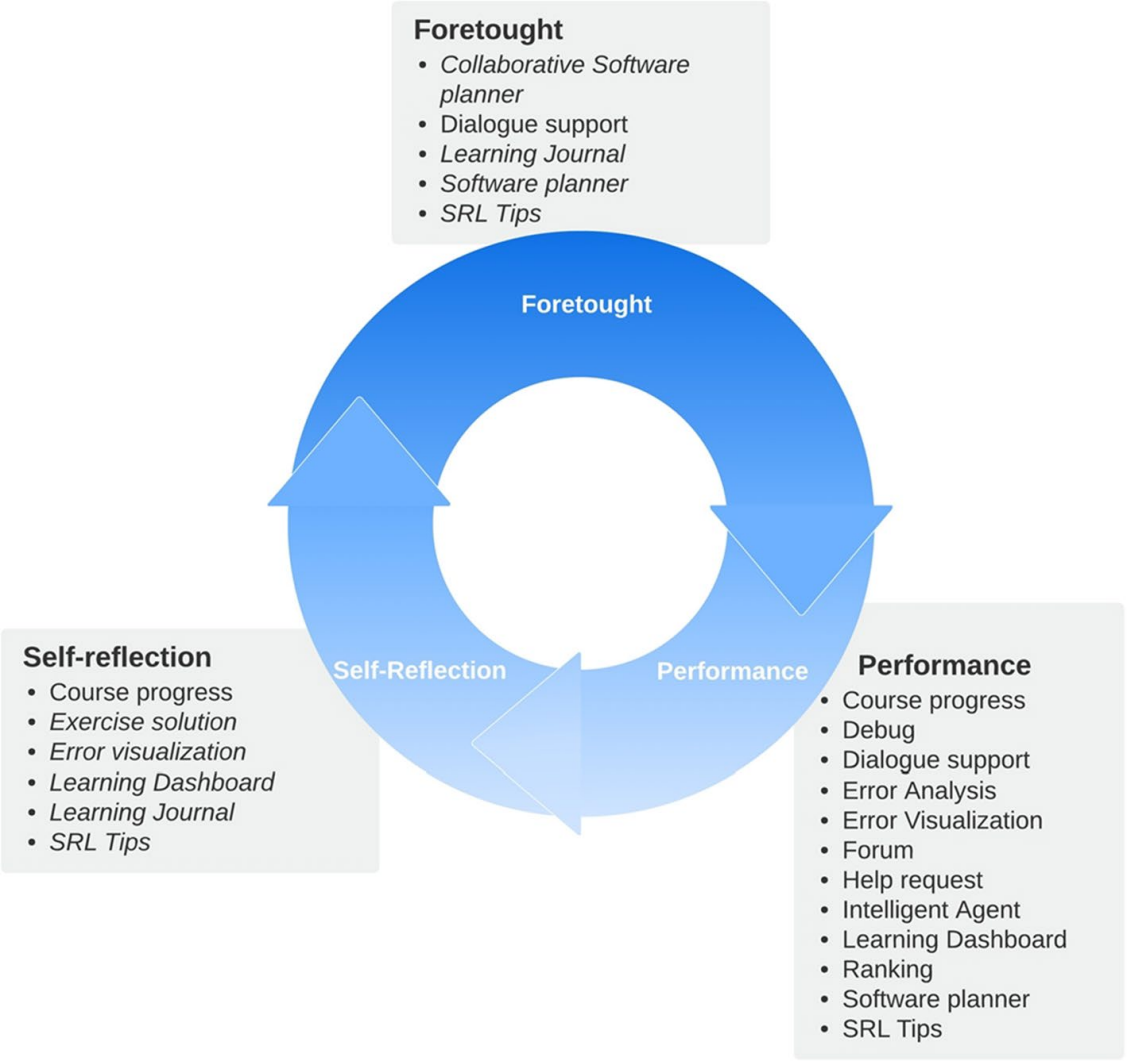
Scaffolding categorization by Zimmerman’s SRL phases

Table 1 provides an overview of the scaffolds that were integrated into RESPE and their respective focus (SRL or social regulation). The proposed scaffolds were based on Zimmerman’s conceptualization of SRL occurrence in phases. Thus, the objective is to support students’ realization of planning, monitoring, and reflection tasks, as represented in Fig. 1.

Stimuli for social regulation were inspired by the design principles proposed by Järvelä et al. (2014). In particular, the need to foster the realization of regulatory processes that enables students to create an awareness of their own and others’ learning process by making resources available that make it possible for students to share and interact.

The scaffolds were presented in two formats: mandatory or manually activated. In the first format, RESPE presented a given scaffold regardless of the student’s wishes. In contrast, in the second format, the use of the scaffolding depended on the student explicitly activating it.

The student manually activated the learning dashboard, forum, debugging, and visualization of the exercise’s solution scaffoldings. The learning journal and software planner had both mandatory and manual usage. For example, after filling out the learning journal (which was mandatory), students could visualize their previous learning journals, allowing a revision of their learning process, a known metacognitive action (Schwendimann et al., 2018). In addition, students could visualize their plan during the code development process. The remaining scaffolds were mandatory.

#### RESPE utilization example

This section provides an example of how the RESPE software was used by a student named Pedro (a fictional name used to protect the student's identity) who belonged to the experimental group. The usage flow depended on whether the scaffolds were mandatory or manually activated. If the scaffolds were mandatory, RESPE guided Pedro on what he should do and in what sequence, whereas if they were manually activated, he could choose to access the features at will.

To investigate the manually activated scaffold usage, we recorded time-stamped logs of student access to them (date and time). This data makes it possible to understand the frequency of use of each separate scaffold as well as the contexts in which this happened. This knowledge was used to build Markov models representing typical progressions within student scaffold usage, as we anticipated that insights from these models would prove helpful in investigating the SRL processes as revealed in previous research (Schumacher & Ifenthaler, 2021). These models describe the sequence of possible events (e.g., the access to specific scaffolding), along with a probability that indicates the chance of transiting from one event to another. By comparing the different behaviors exhibited by students, these models helped us to identify patterns in their use of the RESPE software.

After logging into RESPE, Pedro received an SRL tip through a popup on his screen. For example, “Search for other informational sources, for example, you may use videos on YouTube, lesson materials from other teachers, and websites”. The tip was randomly chosen from a set of 20 sentences prepared by a Ph.D. in psychology with expertise in SRL studies.

**Fig. 2:**
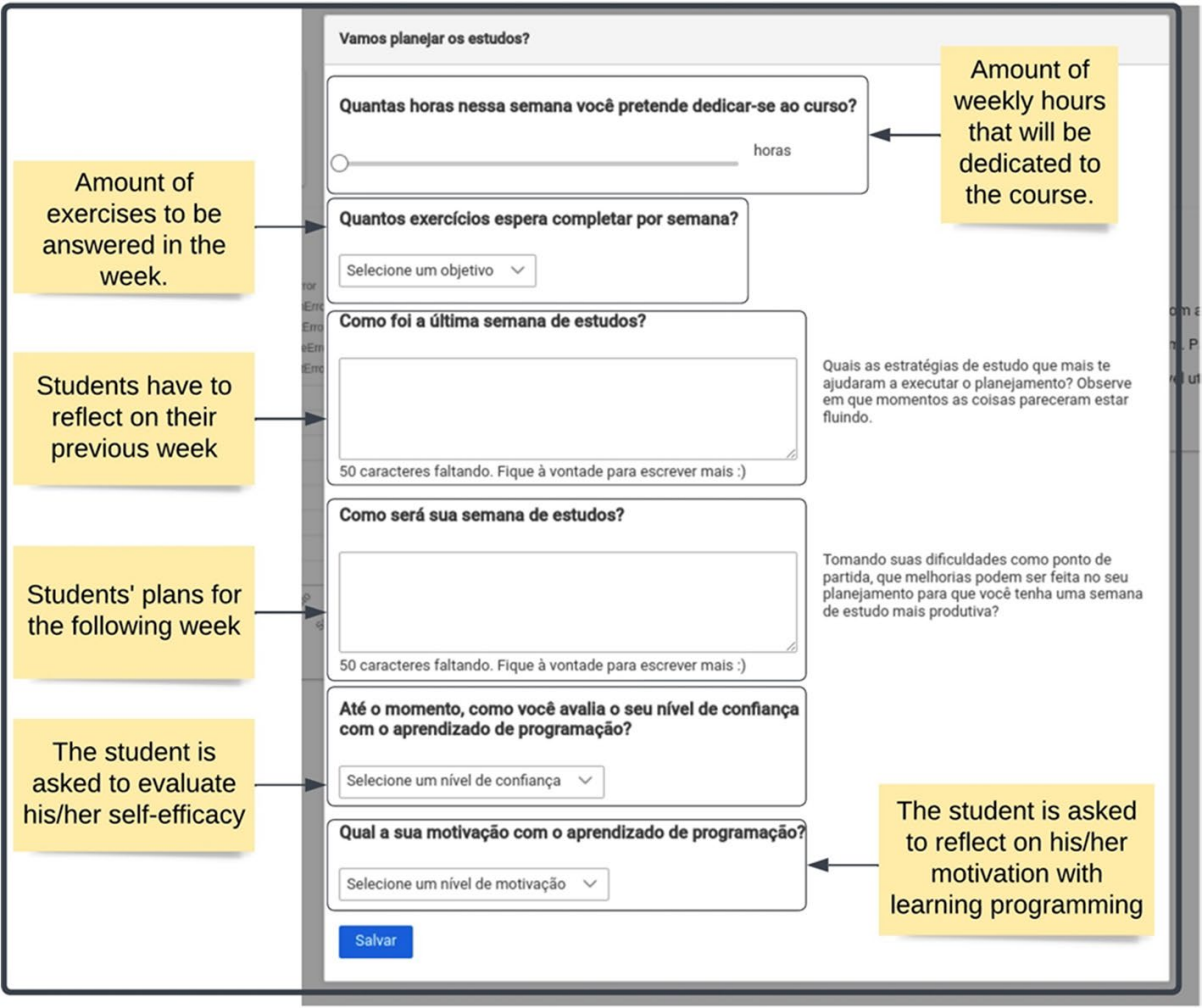
The learning journal interface

After closing the tip and once per week, a learning journal was presented (Fig. 2). This is a question-based scaffold designed to activate behavioral and cognitive processes related to the organization of the study routine and the definition of learning strategies used throughout the week. For example, it had two open-ended questions: “How was your last week of studies?” and “How do you plan your study week to be?”. The aim is to guide students through the forethought and reflection phases of Zimmerman's cycle (Panadero, 2017), which are considered crucial to learning success (Zimmerman & Schunk, 2011).

Pedro also used the learning journal to establish his learning goals, such as the number of exercises and hours dedicated to studying programming. According to Schunk (1990), goal setting should be one of the initial steps taken in the learning process, as it provides a reference about what they should achieve and, as an evaluative criterion, aspects necessary for the regulation of learning.

Previous studies showed that using learning journals proved to be an effective strategy to trigger metacognitive strategies relevant to fostering students’ continuous reflections on their learning processes (Schwendimann et al., 2018).

After filling out the journal, Pedro had several options: access the learning dashboard to visualize his learning metrics, see the top five students in the course using the ranking feature, access the class materials (videos, texts, and exercises), or visualize his previous learning journals.

**Fig. 3:**
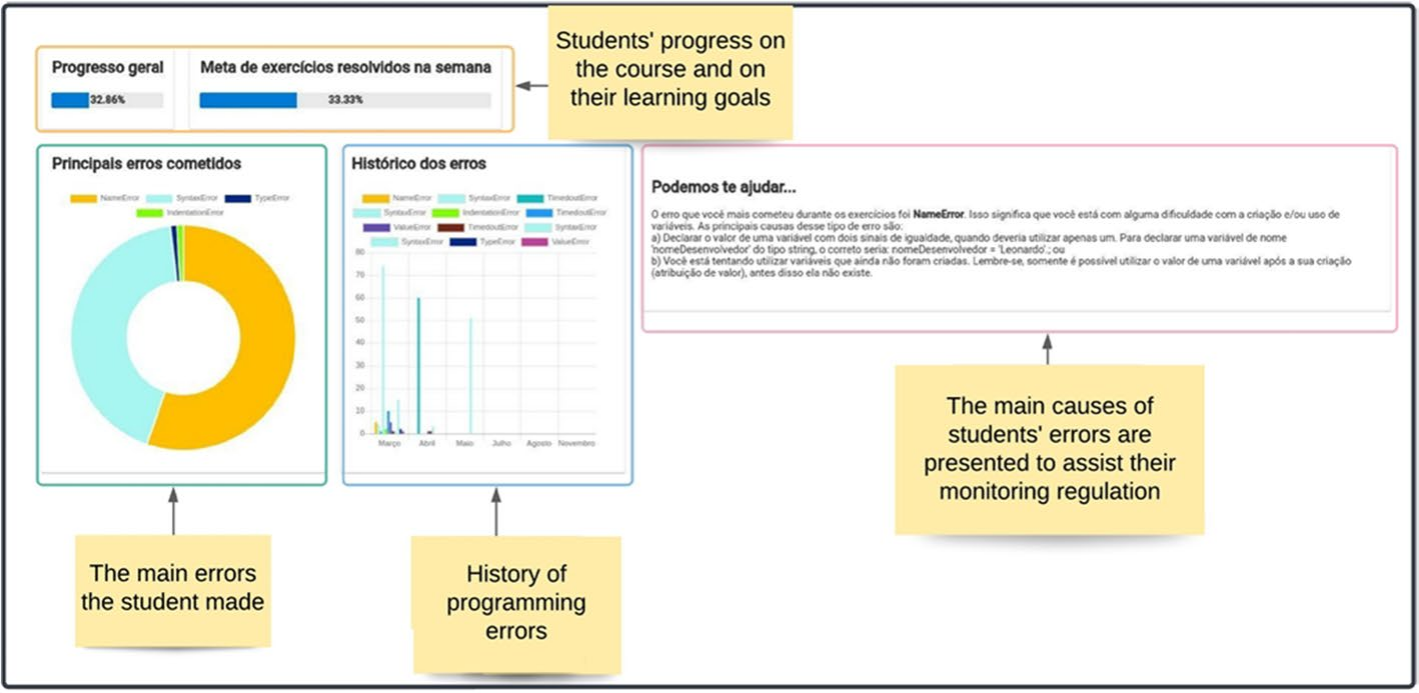
The learning dashboard interface. Several learning metrics were presented using graphics and texts

The learning dashboard design (see Fig. 3) was based on insight into the regulatory mechanisms of metacognitive monitoring, which are expected to improve students’ learning processes by providing them with opportunities to develop an awareness of their academic metrics and to self-evaluate their work (Sedrakyan et al., 2020). In addition, by allowing students to visualize their own data, RESPE provides an Open Learner model (Schumacher & Ifenthaler, 2021), which has proved to be a practical feature to support SRL (Chou & Zou, 2020; Hooshyar et al., 2020).

The ranking feature was highly utilized, not only by Pedro but also by other students. This feature was designed to foster extrinsic motivation, an important factor for enabling students to self-regulate (Moos, 2014). However, this is not necessarily a consensus view, as researchers present divergent opinions about using extrinsic motivational stimuli, with some pointing to its importance in conjunction with intrinsic motivation (Kong et al., 2012) and others arguing the adverse effects of its use (Williamson, 2015).

The access to the course materials featured a list of subjects and a progress bar that showed Pedro’s performance on each. Upon selecting a topic, a list of the videos, texts, and exercises was displayed. Different colors were used to indicate the exercise´s competition (black for unanswered, green for correct and red for incorrect answers), allowing for monitoring his performance on each. Providing students with information about their progress is essential for engagement in self-regulation (Seufert, 2018).

Pedro received regulatory support during programming exercises through the use of scaffolds that were each categorized either as general-purpose and programming-specific. Although regulatory strategies tailored to programming are crucial in this learning domain (Falkner et al., 2014), most of the interventions identified in existing literature have focused mainly on general self-regulatory aspects (Silva et al., 2021a).

**Fig. 4:**
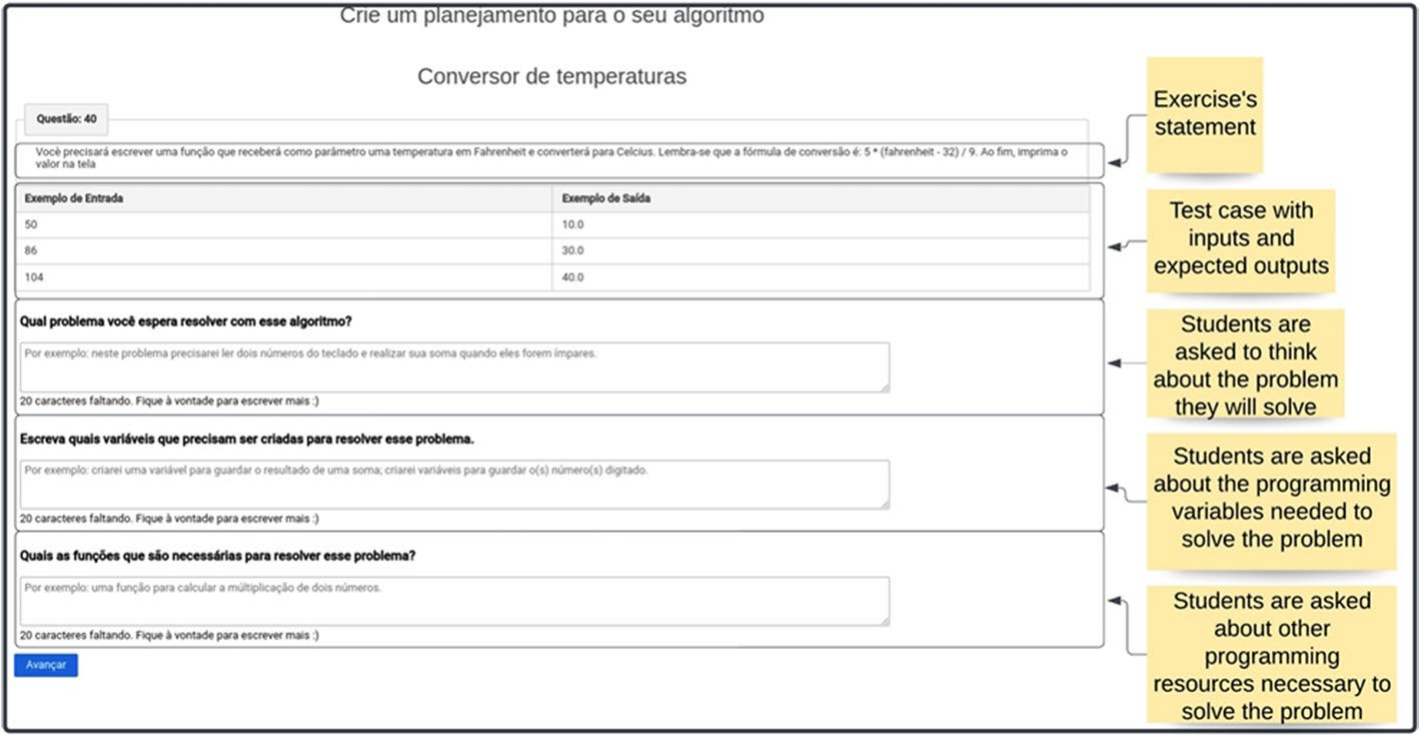
The software planner interface was designed with questions designed to guide students’ problem-solving processes

The first support for programming exercises was the software planner (Fig. 4), a question-based scaffolding designed to guide students in planning their coding process. The following questions were presented: “What is the problem you have to work on in this exercise?” along with personalized questions according to the exercise’s characteristics. For example, if it is an exercise that requires the student to use conditionals,^2^ then the following question is presented: “What are the conditionals required in this exercise?”. Although task planning is crucial, this is often neglected by students (VanDeGrift et al., 2011), resulting in difficulties in program creation (Arakawa et al., 2021).

The pedagogical design of the software planner was influenced by Winne and Hadwin’s definition of task conditions, particularly the concept of instructional cues (Winne, 2017). In this sense, the questions act as guidance to support students’ cognitive processing and foster their SRL. Besides supporting the planning of code creation, the software planner could also be used as a monitoring tool during task execution. However, this was not mandatory and depended on students’ explicit intentions.

**Fig. 5:**
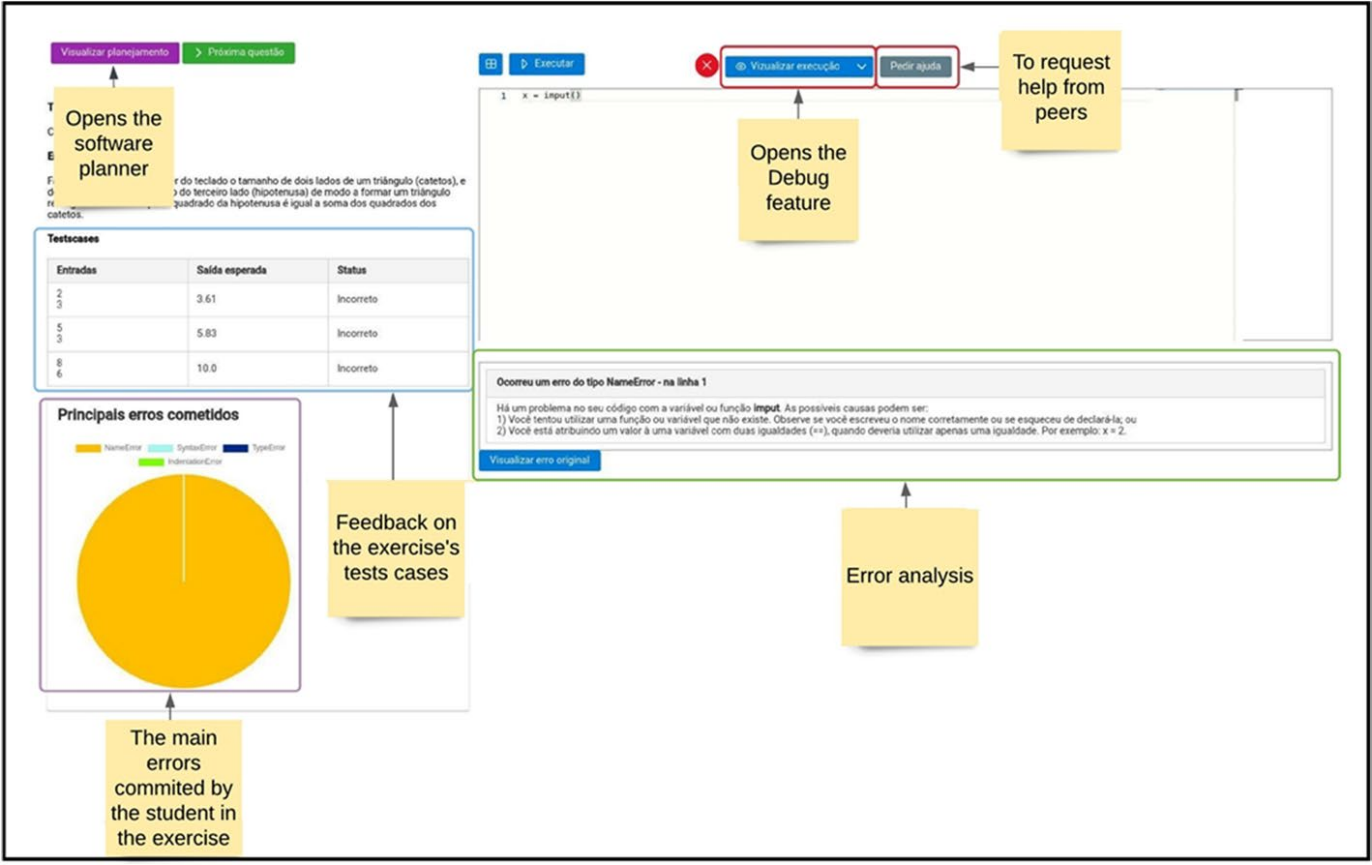
The programming editor interface where students answered the exercises

Answering the software planner was a prerequisite for accessing the programming editor interface, which houses the code development (Fig. 5). During this process, multiple instances of feedback related to the task’s progress and the main errors committed are presented. According to Winne et al. (2013), feedback is essential for students to create an awareness of their performance and monitor their learning.

The RESPE platform collected multiple data during Pedro’s code development process. This data was used for multiple purposes, such as providing personalized feedback and guidance. For example, Pedro committed several programming errors during code development, which is common for novices (Robins, 2019). This data was used to build the error visualization scaffold (a pie chart presented in Fig. 5) that summarizes the main errors committed during the exercise. He could use this information to build awareness about his difficulties.

The aforementioned data were also used by the error analysis scaffolding, an intelligent agent that provided personalized guidance messages and tips on certain learning strategies that could be used to overcome programming errors. In addition, the agent used Pedro’s errors to suggest complementary learning materials that might interest him.

The agent actions were inspired by the concept of coregulation, where the regulatory process of one individual is guided by another (Hadwin & Oshige, 2011). In a classroom context, co-regulation can be accomplished by another student or a teacher. However, since human support is not always available, we sought to stimulate co-regulation using an automated computational interface.

Pedro could use the Debug feature to overcome his programming errors. It is a manually activated resource that presents additional data about the student’s code as it executes, such as the values of variables as well as pointers into the programming execution flow. This data is hidden during typical code execution in order to avoid cognitive load but is relevant when the intention is to trace the execution for the purpose of debugging. From a regulatory perspective, this knowledge is crucial because it reveals how the students’ code behaves, which can assist in their metacognitive monitoring. For example, Pedro might have had some hypotheses about his code execution flow and used the debug feature to verify them. Thus, this knowledge can be used by Pedro to adjust his cognitive strategies accordingly.

To support Pedro´s awareness of his task progress, the programming editor interface provided notifications about his program correctness, as calculated from a set of test cases associated with each exercise.^3^ This support is particularly useful in programming education because students often have difficulties in monitoring their progress, sometimes believing they have a correct or near correct solution when, in fact, they do not (Prather et al., 2018). In addition, the explicit knowledge about the evaluative criteria is fundamental to cognitive processing (Greene & Azevedo, 2007).

If the scaffoldings previously discussed were insufficient, Pedro could still view the exercise’s resolution or request help from his peers. In the first case, a reference program written by the course instructor was presented, which aims to assist students in understanding how the exercise could be solved. To avoid abuse, the student was warned that the exercise would not be scored if the feature was used. However, students who completed the exercise without using this feature could view the teacher’s answer afterward without receiving any penalty. The objective is to stimulate Pedro to compare his work, discover new possibilities for solutions, and self-assess his performance, all of which are crucial aspects of SRL (Winne, 2017).

Pedro could also request help from his peers while solving the individual exercises. By clicking on a button in the programming editor interface, a post in the forum was created, including a copy of Pedro’s code and a message indicating the need for assistance to overcome the problem. Pedro could also use the forum to interact with others, self-initiating a conversation, or respond to other user´s help requests. A forum represents a relevant resource for fostering students’ interactions and co-regulatory episodes (Chan, 2012).

Once per week, Pedro participated in a synchronous class and worked in dyads or triads during programming exercises. To achieve productive collaboration, Pedro and his peers had to regulate themselves (self-regulation) and jointly establish shared goals and strategies by performing social regulation (Hadwin & Oshige, 2011). This is a continuous and laborious process in which students have to engage, negotiate, select learning strategies, request help from peers, and adapt learning objectives, among others, to achieve a collective learning objective. In many cases, these regulatory processes do not emerge naturally, and students need support (Miller & Hadwin, 2015).

The support for collaborative activities was provided in the collaborative software planner feature, which comprised multiple scaffolds. The collaborative and regulatory process orchestration was mediated using a scripting approach, a technique that organizes the collaborative process as a sequence of pre-defined phases. Structuring the collaborative process can be helpful, as research shows that this process often does not occur on its own (Rummel & Spada, 2005). This technique has been extensively used in the context of CSCL for programming education (Silva et al., 2021b), and previous studies also show that this type of orchestration of collaborative activity can be a facilitator of social regulation (Miller & Hadwin, 2015).

**Fig. 6:**
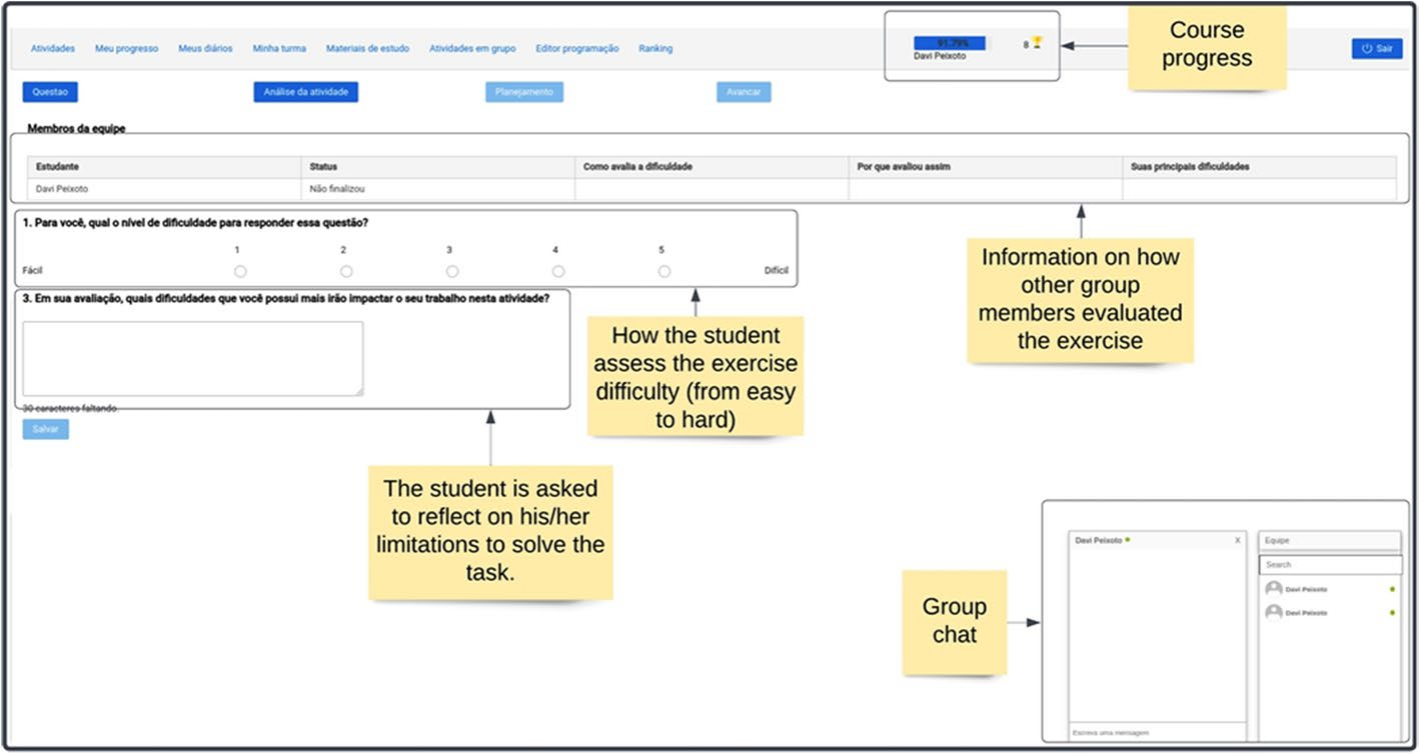
The collaborative software planner interface

In RESPE, the collaboration was structured in a sequence of actions Pedro and his group members were expected to perform. First, they were asked to externalize their knowledge about the task and self-evaluate their capabilities (Fig. 6), known SRL resources (Zimmerman & Schunk, 2011). To accomplish this, Pedro was asked to evaluate the task difficulty using a 1 to 5 scale (from easy to difficult): “For you, how difficult is it to answer this question?”. He was also asked to explain his answer and discuss difficulties he anticipated that could impact the exercise resolution process. In reflecting on the task’s difficulty, Pedro was required to establish criteria to evaluate the task, which involves both cognitive and metacognitive processes. Other group members also answered the same questions, and the data was shared among them. Creating awareness of others’ capabilities and limitations is a crucial aspect of social regulation (Järvelä et al., 2016) and essential to generate co-regulation episodes throughout the activity (Chan, 2012; Järvelä et al., 2019).

**Fig. 7:**
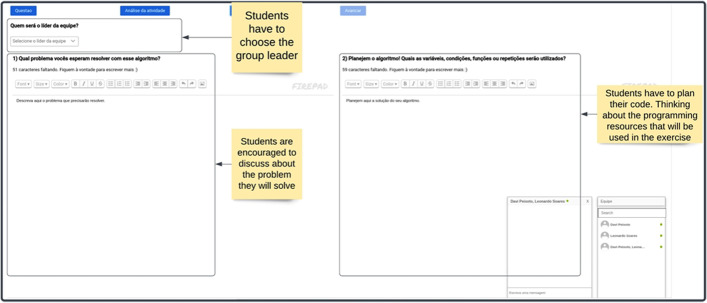
The second interface of the collaborative software planner

In the second part of the collaborative software planner, students collaboratively define tacit strategies to solve the programming problem (Fig. 7). Pedro and his peers had to choose one team leader, crucial to orchestrate activities execution (Schellens et al., 2007). The group members were also requested to discuss their task understanding: “What is the problem your team is going to work on?” (Järvelä & Hadwin, 2013). Then, another question was presented to guide the group plan of the programming features necessary to solve the problem. These questions were answered in a collaborative editor that shared its content in real-time among group members.

Access to the programming editor was only available after answering all the questions, and only the group leader had permission to indicate that the collaborative planner was completed. Upon doing so, all members were redirected to the program editor interface. The programming editor for the collaborative activities was similar to the one shown in Fig. 5. However, it included collaborative features, such as real-time code updating, allowing all group members to visualize the same programming code. In addition, group members had access to a chat room where they could interact textually or using voice.

As group work quality is often lower due to poor interaction during collaborative learning (Hadwin & Oshige, 2011), an intelligent agent (named dialogue support) was provided to stimulate productive interactions between students. This resource monitored Pedro’s and their peer’s interactions in the chat. In their absence, different messages were presented, such as: “Your participation in the group is essential to achieve a good result”, “Don’t forget to collaborate with your colleagues!”, and “Are you having difficulties? Share with your friends. They can help you!”. Furthermore, tips for improving the group’s problem-solving capabilities are presented: “Do you remember having solved a problem like this? It’s the first step to knowing how to solve the current problem.”.

Students in the experiment who did not receive social regulation support did not have access to the collaborative planner and received suggestions from the dialogue support. However, their programming editor had basic collaborative features (e.g., real-time editor and chat).

## Method

An experiment was performed to evaluate the RESPE’s effectiveness.

### Hypothesis definition

Based on the understanding that regulation of learning is crucial for learners of programming and that it can be stimulated (Garcia et al., 2018), we raised three hypotheses: H_1_: Students’ course grades in the experimental groups would outperform the students in the control group; H_2_: The performance in collaborative activities of students who received social regulation support would outperform the students who did not receive this support; and H_3_: Students’ SRL scores in the experimental groups would outperform the students in the control group.

### A priori power analysis

The sample size necessary for the experiment was calculated through an a priori power analysis. One of the approaches uses an average effect size from prior research in a similar context to determine the sample size (Kang, 2021). However, due to the lack of this data in our context, we had to calculate this effect size through a random-effects meta-analysis.

Three data sources were used, the findings from our previous systematic literature review on interventions grounded on the regulation of learning theory in programming education (Silva et al., 2021a) and the findings from two other systematic reviews (Garcia et al., 2018; Loksa et al., 2022). The inclusion criteria were the experiments using interventions grounded on the regulation of learning constructs in programming education. In addition, they had to include a control group and measure programming learning as an outcome.

A total of seven studies^4^ were included in the analysis resulting in a total sample size of 844 students. The findings point to an average effect of 0.59 with a confidence interval (C.I) of [0.28, 0.89], which is consistent with the effect size of SRL interventions in other domains (Dignath & Büttner, 2008; Ergen & Kanadli, 2017; Zheng, 2016a).

The power analysis was performed using the Gpower software (Kang, 2021), for 95% power, which is a typical and accepted level in statistical analysis (Lakens, 2013). The computation was performed for ANCOVA with three groups and one covariate, following our experimental design specifications and indicated the minimum sample size of 41 students.

### Pedagogical context

A mid-sized public university in an urban area in Brazil offered online introductory programming courses aimed at people with little or no programming background. Each course lasted ten weeks with a 40 h duration with synchronous and asynchronous classes. The course took place during the COVID-19 pandemic, had no subscription fee, and the professor was the first author of this study.

The syllabus was based on the ACM Curricula for fundamental programming concepts, encompassing variables, input/output, conditionals, loop structures, and functions,^5^ using the Python language.

Students had asynchronous access to the recorded video materials consisting of programming classes and exercises, which were made available weekly following the presented syllabus sequence. All course content was released in Portuguese, the student’s native language.

Programming practice took place in the RESPE learning platform. The programming exercises were divided into individual and collaborative activities, and in both of them the expected outcome was a working program based on the requirements presented in the exercise. A total of 70 exercises (13 from variables, 24 from conditionals, 21 from loops, and 12 from functions) were provided with varying difficulties and were organized according to Bloom’s Taxonomy (Thompson et al., 2008). Collaborative activities consisted of eight exercises (two from variables, three from conditionals, two from loops, and one from functions). In order to foster reproducibility, the exercises were translated to English and are available in our Open Science Repository: https://osf.io/tu47x/?viewonly=aab2c35ee94d41b1abaf5ececea3f0dd.

Each exercise included a statement describing a problem and a set of test cases. The exercises were made available following the course’s calendar and syllabus sequence, and students were able to work on them until the end of the course. There was no limit to the number of times the students were allowed to test their programs during the same exercise.

Collaborative activities occurred during one and a half hours of synchronous meetings that were scheduled weekly. In these meetings, the students asked questions and worked collaboratively (dyads or triads randomly drawn at each meeting) on one or two programming exercises. The exercises were related to the content addressed in the previous week, and they did not have the teacher’s support in carrying them out. The correctness of the collaborative activities was measured using the corresponding test cases, and all students in the same group received the same score. The student’s participation in the synchronous meetings was optional and did not add to their final grade in the course.

The RESPE automatically calculated students’ course grades based on the correctness of the individual exercise’s test cases. Those who obtained a grade equal to or greater than 70 (out of 100) obtained a certificate for the course.

### Data collection and analysis

The data collection and analysis procedure were directed toward testing the research hypotheses.

#### RQ1) Do students who received regulatory support achieve higher performance in programming learning compared to students who did not receive support?

The RESPE calculated the students’ final course grades based on program correctness and was used to test H_1_: *Students’ course grades in the experimental groups would outperform that of the students in the control group*.

The hypothesis H_2_: *The performance in collaborative activities of students who received social regulation support would outperform the students who did not receive this support*, was tested using the group responses to the collaborative exercises. A binary classification (correct or incorrect) was used, based on the program’s correctness according to the specified test cases and whether the solution was submitted on schedule.

#### RQ2) Do students who received regulatory support achieve higher performance in developing regulatory skills compared to students who did not receive support?

Student regulatory skills development was measured using a Portuguese version of the Motivated for Learning Strategies Questionnaire (MSLQ) (Pintrich & Groot, 1990), with a five-point Likert scale. The instrument was translated into Portuguese by the authors of this research.^6^ The Cronbach alpha for this instrument was 0.81 [95% Confidence Interval (C.I.) of 0.75, 0.88], demonstrating good internal consistency (Cortina, 1993) and in consonance with other MSLQ translations (Jakesova & Hrbackova, 2014).

The original instrument stands out as one of the most widely used to measure self-regulation aptitude in different contexts, including programming education (Loksa et al., 2022). The questionnaire is divided into three constructs, motivation, cognition, and metacognition. Only the cognitive and metacognitive scales were used because we focused on stimulating these areas. According to the MSLQ authors, it is possible to use parts of the instrument (Pintrich & Groot, 1990), which has also been done in several other studies (Prather et al., 2020).

Students’ MSLQ scores were used to test the H_3_: *Students’ SRL scores in the experimental groups would outperform the students in the control group*. The cognitive strategies mentioned in the instrument relate to students’ ability to organize their learning materials, focus and comprehend the relevant parts of a text, perform annotations to organize learning ideas, and others. The metacognitive strategies focus on students’ ability to monitor and plan their learning and overcome difficulties.

A typical procedure to assess SRL development is to use a pre, and post-test design (Loksa et al., 2022), which was performed in this investigation. In this regard, the pre-test could be used as a covariate to assess differences in the post-test across students in different experimental conditions.

### Experimental design

**Fig. 8:**
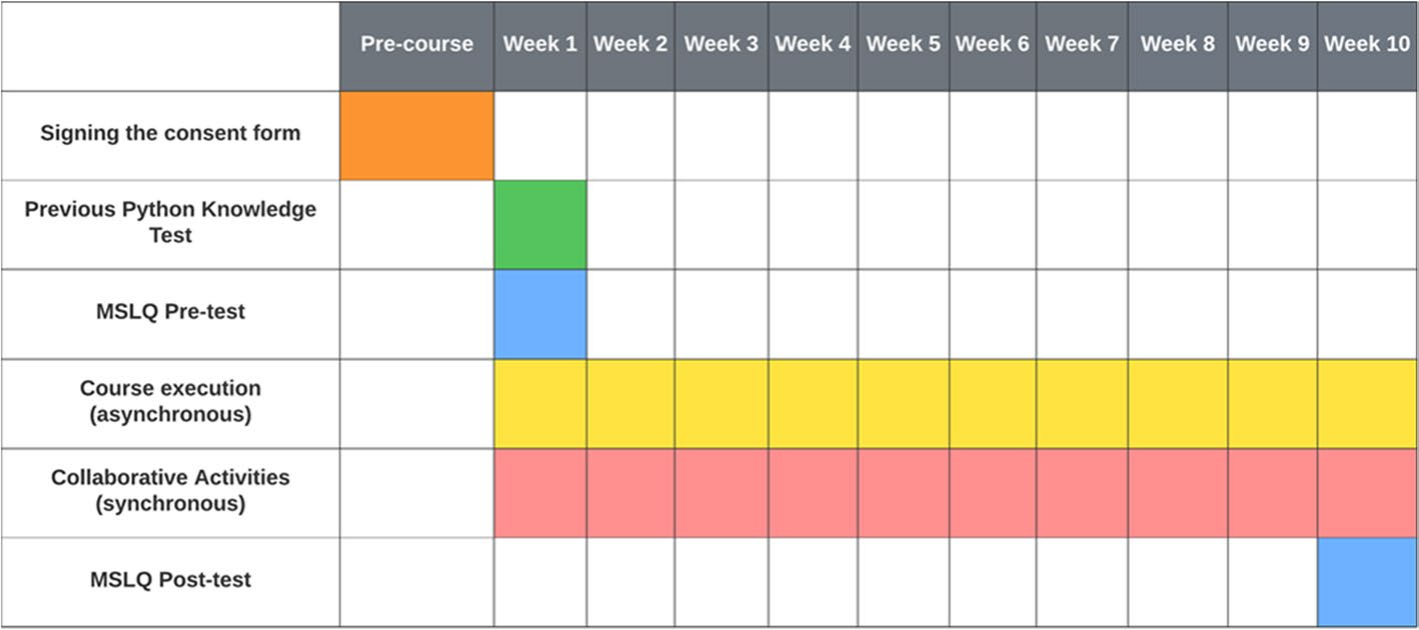
Presentation of the different phases carried out throughout the course and the week they occurred

Figure 8 presents how the experiment was structured. This study is a single-blind experiment because the participants did not know to which group they belonged, reducing the risk of detection bias. On the other hand, the course professor knew which group his students belonged to.

The independent variable was the learning platform RESPE, with three levels: two experimental conditions, named ExpSRL and ExpSRLSharedReg, and one control group (C.G.). Students in the ExpSRL group received support for SRL, whereas the ExpSRLSharedReg group received both SRL and social regulatory support. The learning platform used by the control group had no regulatory support, consisting only of the course materials and the programming editor. Students in the C.G. viewed the standard Python interpreter messages as if they were in a traditional programming editor.

Three dependent variables were measured in this work: i) students’ final course grades, ii) the number of collaborative exercises correctly and delivered on schedule, and iii) MSLQ scores.

Confounding variables known to affect the learning process were controlled, such as course duration, exercises, professor, and learning material (Ewert & Sibthorp, 2009). Therefore, these variables were the same for all the groups.

### Sample

The sample consisted of students enrolled in three programming courses offered sequentially. The courses were offered with a one-month interval between them and took place during the COVID-19 pandemic. The experimental conditions of each course (ExpSRL, ExpSRLSharedReg or C.G.) were randomly selected before the start of the first course. The students in one course were all in the same experimental condition.

One hundred and twenty-three students enrolled in the courses, and 52 dropped out. One of the biggest challenges of online courses is the high number of dropouts, and our rate is similar to those of other online courses (Bawa, 2016). Table 2 presents the baseline characteristics of all participants, including those who dropped out and were lost to follow-up. The last column (remaining participants) reports the data from students that participated in the experiment (*n* = 71).

**Table 2:** Baseline characteristics of all participants

Baseline variables	All participants			Participants dropout			Remaining participants		
	E1	E2	C.G	E1	E2	C.G	E1	E2	C.G
Age – mean (SD)	31.25 (12.27)	23.29 (9.61)	27.15 (12.11)	27.07 (7.59)	22.94 (8.78)	27.85 (9.45)	34.52 (14.4)	23.48 (10.15)	26.45 (14.51)
Female	12	20	10	10	8	6	2	12	4
Male	19	31	31	4	10	14	15	21	17

*E1* ExpSRL group; *E2* ExpSRLSharedReg, and *C.G.* Control group

At the beginning of the course, students were informed about the data collection procedure, and the sample consisted of students who signed the consent form. There were no incentives offered for participation in the study.

**Fig. 9:**
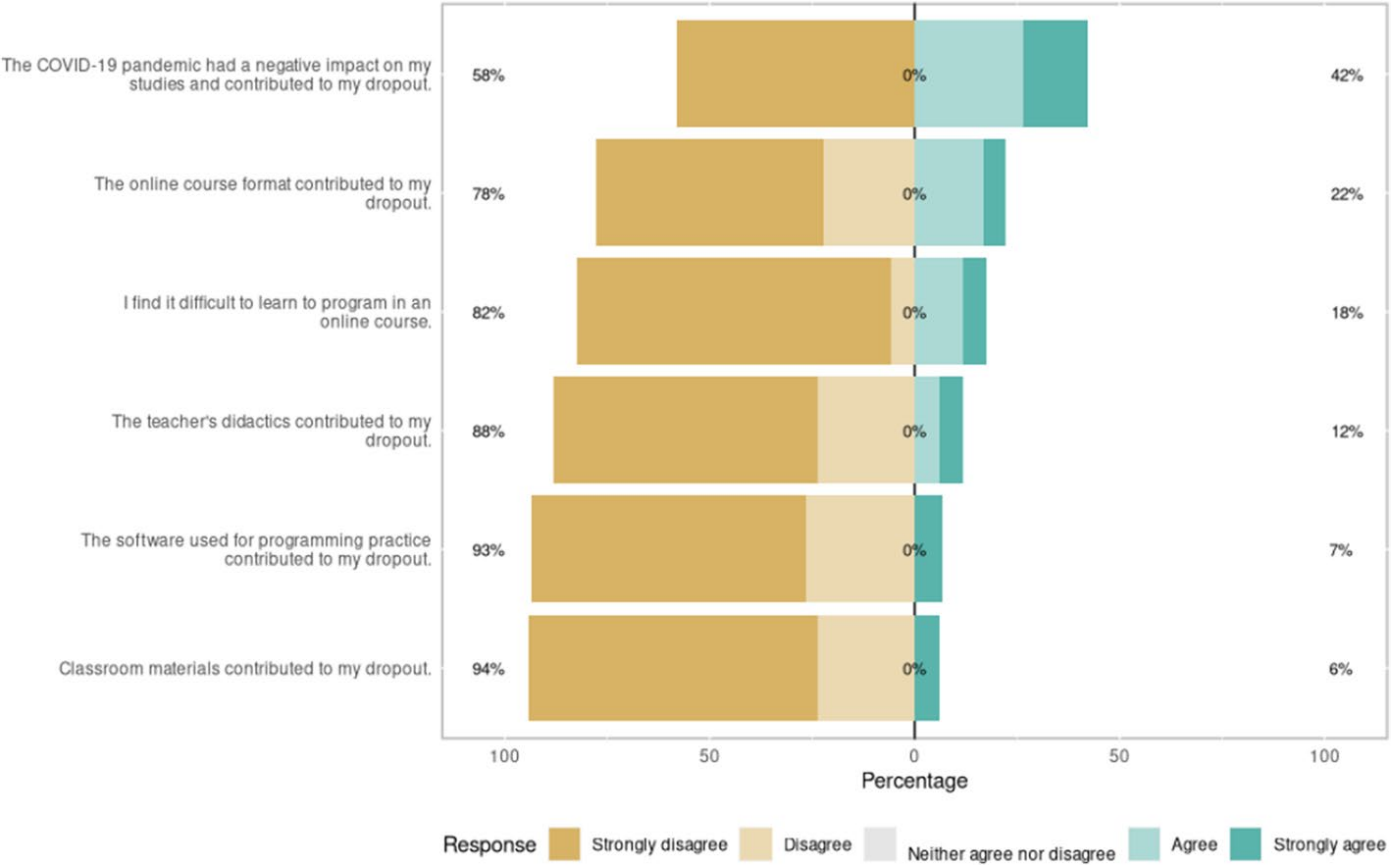
Likert responses on the dropout questionnaire

Students lost to follow-up were contacted through email to identify the dropout causes and their relationship with the experiment. An anonymous questionnaire was sent featuring one open-ended question, (“Please specify the causes that led you to drop out of the course.”), and six items describing the multiple factors that may have affected the student, which were answered with a five-point Likert scale from “I completely disagree” to “I completely agree”. Items and responses are presented in Fig. 9.

Thirty-five percent of dropout students answered our emails. From the open-ended question, four main themes emerged: lack of time to balance the course with other activities (65%), problems with equipment, such as lack of Internet, computer, among others (20%), programming learning difficulties (10%) and criticism of the format of virtual classes (5%). Responses show that most of the dropouts happened for reasons unrelated to the experiment.

Pearson’s chi-square test was performed to identify the association of students’ dropouts with the experimental condition they belonged to, which helps to evaluate if dropouts were higher in certain experimental groups. No statistically significant difference was identified (*p* = 0.64).

A series of statistical analyses were performed to assess eventual differences that might exist across the samples’ characteristics in the experimental conditions. A Pearson’s chi-square test revealed no differences (*p* = 0.12) regarding gender frequency. A Kruskal–Wallis test was performed to determine differences between the medians of the age of the participants. A post hoc analysis using the Dunn test with a p-value adjusted using the Holm-Bonferroni method revealed that the median age of students in the ExpSRL group differed from those in the ExpSRLSharedReg, which was the only statistical difference.

Lastly, before the start of the courses, students answered a programming test to assess their previous Python knowledge. Students presented an average score of 1.31 (out of 10) with a 2.41 standard deviation, demonstrating having little to no knowledge of this programming language. A Kruskal–Wallis test was performed to assess differences in the medians across the three experimental groups (ExpSRL, ExpSRLSharedReg, and C.G.), and no statistically significant difference was identified (*p* = 0.63). Therefore, there was no difference between the groups regarding previous Python knowledge.

## Results

### RQ1) Do students who received regulatory support achieve higher performance in programming learning compared to students who did not receive support?

The RESPE’s effectiveness in fostering learning of computer programming was assessed in this research question.

#### Hypothesis 1

This section presents the test of the null hypothesis of *H*_*01*_*: Students’ course grades in the experimental groups would not outperform that of the control group students*. The mean and standard deviation of students’ course grades are presented in Table 3.

**Table 3:** Final course grade mean and standard deviation grouped by experimental conditions

	ExpSRL	ExpSRLSharedReg	C.G
Sample size	17	33	22
Mean (SD)	69.3 (31.24)	63.13 (32.08)	39.05 (28.4)

A one-way ANCOVA was performed to compare the effectiveness of the RESPE while controlling for students’ previous programming knowledge scores. Prior research examined the influence of previous programming knowledge on learning achievement and found that it correlates with course grades (Campbell et al., 2016), leading to our decision to choose this variable as a covariate.

The homogeneity of variance was assessed using Levene’s test (F = 0.11, *p* = 0.89). A significant difference in mean course grades was identified [F(2, 68) = 5.607, *p* = 0.006]. Post-hoc analysis performed using Tukey’s HSD showed a significant difference between ExpSRLSharedReg and C.G. (*p* = 0.02); and ExpSRL and C.G. (*p* = 0.008). No statistical difference was found between ExpSRLSharedReg and ExpSRL groups. The estimated marginal means showed that students in the ExpSRL group achieved the highest grade (M = 72.5), followed by ExpSRLSharedReg (M = 62.3) and C.G. (M = 38.0).

Based on this result, we conclude that students who received the regulatory support achieved better performance when compared to the control group, regardless of the type (SRL only or SRL and social regulation). Thus, the null hypothesis H_01_ was rejected (α = 0.05).

The effect size was measured using the partial eta-squared and calculated with the R package named *effectsize*, resulting in an effect of 0.18 [95% C.I:[0.01, 0.34], which could be interpreted as a medium effect in the educational domain (Richardson, 2011).

#### Hypothesis 2

Our null hypothesis of *H*_*02*_*: The performance in collaborative activities of students who received social regulation support will outperform the students who did not receive this support*, was tested.

A Pearson’s Chi-Squared test was conducted to assess whether achieving a correct solution in the collaborative exercise was related to the students’ experimental conditions. There was significant evidence of an association (*χ*^2^ = 9*.*5375*, p* < *0.0*1). A post hoc analysis was performed on the residuals of the Chi-Square test, with the p-value adjusted using the Bonferroni correction. A statistically significant difference was identified between ExpSRLSharedReg and ExpSRL; and between ExpSRLSharedReg and C.G. In addition, no statistically significant difference was reported between the C.G. and ExpSRL. Thus, the null hypothesis H_02_ was rejected (α = 0.05), indicating that the performance of the ExpSRLSharedReg group differs from the others. A Cramer’s V effect size of 0.36 was identified (95% C.I. [0.1, 0.58]), which is considered as a medium effect (Sun et al., 2010).

The result above supports the relevance of social regulation support on collaborative tasks. At the same time, it was surprising that the ExpSRL group’s scores were not statistically different from the control group. The interpretation of these results should consider that due to the way performance in collaborative activities was scored, it was not possible to use prior programming experience as a covariate as in the other hypotheses.

### RQ2) Do students who received regulatory support achieve higher performance in developing regulatory skills compared to students who did not receive support?

Students’ SRL skills were measured using the MSLQ instrument. Thus, we performed a statistical analysis to identify differences in their scores across experimental conditions.

#### Hypothesis 3

The null hypothesis *H*_*03*_*: Students’ MSLQ scores in the experimental groups would not outperform the students in the control group*, was tested. Students’ mean and standard deviation on the MSLQ pre and post-test are presented in Table 4.

**Table 4:** Students’ MSLQ mean scores and standard deviation grouped by experimental conditions

	MSLQ mean pre-test (SD)	MSLQ mean post-test (SD)
ExpSRL	3,97 (0.48)	4.0 (0.39)
ExpSRLSharedReg	4,05 (0.38)	3.95 (0.38)
C.G	3.98 (0.51)	4.1 (0.54)

A one-way ANCOVA was performed to compare the effectiveness of RESPE in fostering SRL skills whilst controlling for students’ previous SRL scores. The homogeneity of variance was assessed using Levene’s test (F = 0.23, *p* = 0.79). No statistically significant improvements in the post-test were observed (*p* = 0.32), and the null hypothesis could not be rejected.

## Discussion

Our discussion follows the research questions order.

### RQ1) Do students who received regulatory support achieve higher performance in programming learning compared to students who did not receive support?

Researchers and educators argue that providing regulatory support is crucial to mitigate students’ limitations, leading them to successful learning behavior (Dignath & Büttner, 2008). In addressing this research question, we sought to contribute evidence about the effectiveness of regulatory support in programming education, not only from a self-regulated perspective but also incorporating social regulatory support, which is lacking in existing literature (Garcia et al., 2018).

The programming learning process cannot be disassociated from the ability to use and control mental processes (Loksa et al., 2022). An association between regulatory skills and programming learning performance was identified by previous research (Silva et al., 2021b). In addition, the lack of SRL skills was associated with difficulties in completing programming exercises and low programming performance (Falkner et al., 2015). This evidence highlights the importance of regulation of learning to programming education.

#### Hypothesis 1

The statistical analysis using students’ final course grades revealed that regardless of the regulatory support (individual or individual and social regulation combined), students in the experimental treatment achieved higher final course grades than the control group. This result corroborates previous findings that providing regulatory scaffolding is an effective strategy that can foster improvements in programming learning (Prather et al., 2020).

**Fig. 10:**
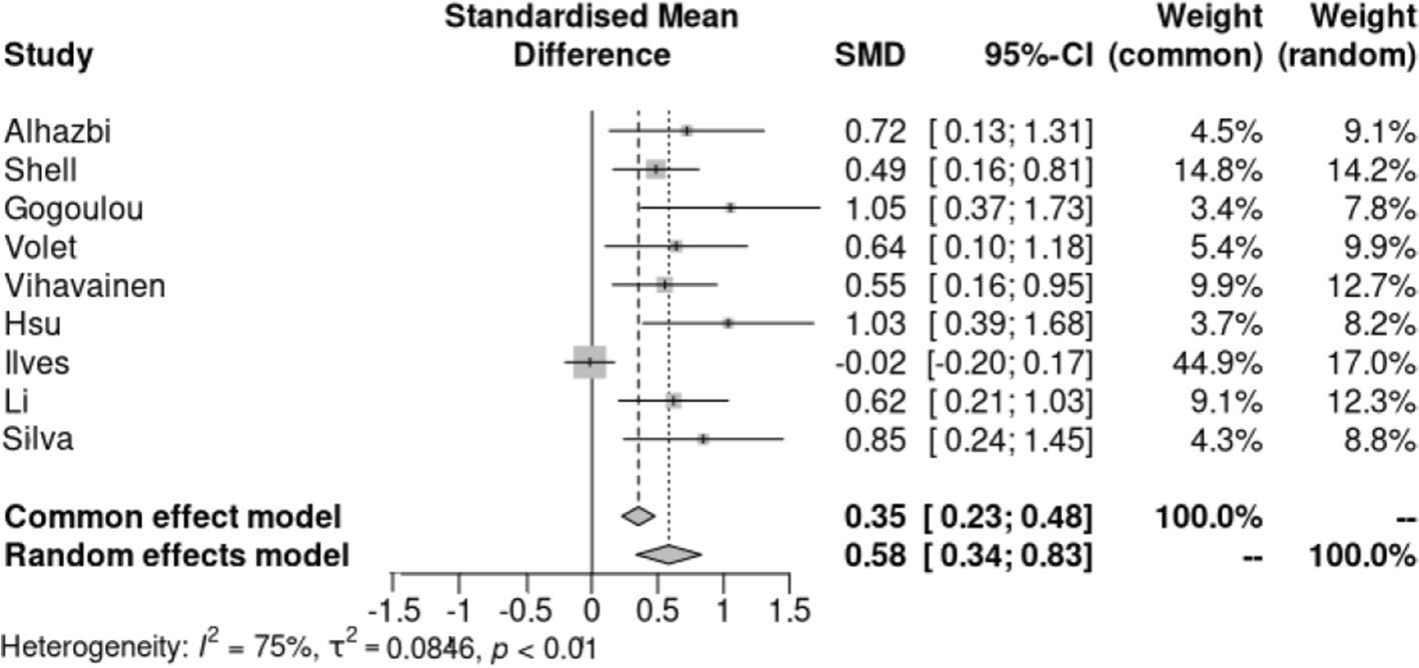
Forest plot with the effect sizes from the studies analyzed in the meta-analysis

The forest plot presented in Fig. 10 compares our result with the findings from the meta-analysis. Our study had the third-largest effect size, corroborating the relevance of our proposition.

With the exception of Ilves et al. (2018), the interventions analyzed in the meta-analysis promoted a positive impact on learning gains, which supports our previous argument. Notably, two of the interventions with the largest effect sizes incorporated collaborative processes, underscoring the importance of this approach for enhancing learning outcomes.

To gain a deeper understanding of the mechanisms behind the regulatory interventions discussed, future research should investigate the specific factors that contribute to their success. One area that requires further exploration is the understanding of how each scaffolding contributes to the final result, as this knowledge is currently limited (Silva et al., 2021a).

In our study we identified some issues related to students’ use of the software that could influence the design of future scaffolds. For example, some students had the perception that they did not need regulatory support (e.g., they did not plan their program development and assessed that this process was not necessary). This finding indicates that providing regulatory support alone is insufficient to assure its use and effectiveness. Therefore, it is crucial to balance the provided support with students’ needs and perceptions about the importance of using them.

In addition, there was also a challenge in seamlessly incorporating regulatory scaffoldings into a programming learning environment. Program development is known to be a process that demands the use of multiple mental resources (Prather et al., 2020), and the impacts of incorporating scaffoldings that stimulate these resources simultaneously are unknown. Thus, we argue the importance of studying this phenomenon from the Cognitive Load Theory perspective (Mutlu-Bayraktar et al., 2019), as providing regulatory support could increase students’ cognitive load (Shin & Song, 2022). Nevertheless, how regulatory support influences cognitive load and its impact on learning still needs further investigation.

#### Hypothesis 2

Students often have difficulties performing group tasks as, in many cases, collaboration does not occur naturally or satisfactorily (Malmberg et al., 2017). Researchers argue that the lack of social regulation among students is one of the causes of this problem (Järvelä et al., 2019).

Hadwin et al. (2017) discussed multiple theoretical perspectives for incorporating social regulation support in CSCL environments as a strategy to mitigate the aforementioned issue. At the same time, there is a strong criticism regarding the limited number of studies designed with this intention (Lin, 2018), such that our understanding of how to effectively design learning environments with social regulatory support is likely too limited (Panadero et al., 2015). Thus, our work aims to provide new empirical evidence on the impact of SRL and social regulation support on achievement of collective learning. By addressing this research gap, we can better understand the potential of social regulatory support to enhance collaboration and improve educational outcomes in CSCL settings.

Providing only SRL support did not seem to be enough for students to achieve improvements in collaborative activities, as their performance did not differ from the control group. This is an interesting finding, and a possible explanation is that collaborative learning demands specific actions that, in many cases, do not occur spontaneously, even by self-regulated learners (Järvelä et al., 2019).

On contrary, social regulatory support seemed to be especially important in collaborative activities. A limitation of our study is that we do not know which social regulatory scaffolds were more effective in this collaborative support. To mitigate this issue, in future work, we plan to leverage an analysis of students’ data (e.g., their chat logs and interactions with the software), which might increase understanding about how social regulation of learning occurs.

### RQ2) Do students who received regulatory support achieve higher performance in developing regulatory skills compared to students who did not receive support?

The evidence about the development of skills related to regulation of learning using CBLEs in the programming education context is still conflicting (Silva et al., 2021b). Part of the research points to improvements in this ability after exposure to the intervention, while others, as in our study, did not find favorable results.

#### Hypothesis 3

Even though no improvements were achieved in the SRL skills development measured by the MSLQ, students who received regulatory support had better learning performance. It is an important finding demonstrating the relevance of providing educational environments with regulatory support. At the same time, it exposes the challenges involved in promoting the development of regulation of learning skills.

Several questions arise from the result above, such as *i)* whether the effects remain without RESPE-mediated support, *ii)* whether the learning gains remain in the long term, and *iii)* whether the regulation of learning skills would be developed after longer exposure using RESPE. A longitudinal study would provide an opportunity to investigate these questions.

The difficulties in developing SRL skills could be motivated by several factors. It is already understood that SRL depends on several other social and psychological variables (or subprocesses). At the beginning of the SRL research, Zimmerman stated that SRL depends on three factors: personal, behavioral, and environmental (Zimmerman & Martinez-Pons, 1988). The support provided with RESPE lies in this latter category, and while important in the SRL equation, its development should consider other factors.

The effectiveness of regulation of learning support is influenced by variables, such as students’ school level, the intervention focus (e.g., cognition, metacognition, motivation, or others), the presence of collaborative activities, among others (Dignath & Büttner, 2008; Theobald, 2021). In addition to these factors, SRL skills development may require time (Shin & Song, 2022). Therefore, these elements should also be pondered when analyzing the results from multiple studies.

Lastly, the quantitative data alone is limited in revealing behaviors that would increase understanding about how different students’ regulatory processes occur. Thus, in future work, we expect to explore other data sources that were collected, such as students’ chat logs and their time-stamped software usage logs. For example, initial findings indicate that high and low-performers have different patterns of using scaffolds, and that increased access to these features was associated with higher performance in the course.

### Implications for research and practice

This work presents theoretical and practical implications for designing and using computer-based regulatory scaffolding. The design process is expected to contribute to development of new scaffolding, particularly in the area of social regulatory support. Additionally, the study provides evidence that individual regulatory support alone is not sufficient for enhancing collaborative activities. This highlights the critical role of social regulation in supporting collaborative learning.

While the results support previous findings on the effectiveness of regulatory scaffolding in programming education, some questions arise from this finding. For example, does the effect persist without RESPE? Will SRL skills be developed with more prolonged exposure using RESPE? These are expected to be answered in future investigations.

Finally, although the RESPE learning platform was used in introductory programming education, the characteristics of the software may make it feasible in general STEM education. However, this aspect should be investigated in future research.

### Limitations and threats to validity

The results presented in this work should be pondered considering our limitations. First, our educational context is in the field of introductory programming. Thus, the findings might not generalize to related areas, such as data structures or advanced programming courses. In addition, the study occurred during the Covid-19 pandemic, which may have influenced students’ learning behavior.

Regarding the demographic characteristics of the participants in this study, only students’ age in the ExpSRLSharedReg group showed a statistically significant variation compared to the ExpSRL group. The influence that age has on learning programming is still a topic with diverging results (Grover et al., 2016; Quille & Bergin, 2018), but it may have some influence.

RESPE´s scaffolds were limited to cognitive and metacognitive processes. However, the learning process is also influenced by other types psychological processes, such as motivation and emotion, which are important as well (Lacave et al., 2020). In addition, because multiple regulatory features were combined, we are limited in identifying which had the most significant effect on learning achievement.

It is also important to acknowledge the limitations that the chosen measurement formats impose. Although the measurement of programming knowledge using the performance in exercises has been adopted in other studies in the literature (Lei & Mendes, 2021), it might differ from other instruments such as a standardized test. Also, our performance analysis in collaborative activities considered the final product, not the individual contributions in this process. Thus, we emphasize that our data limit the analysis of whether there was an imbalance in student participation. Lastly, using a translated version of the MSLQ might impose psychometric issues. Despite a satisfactory Cronbach alpha, which is in accordance with international literature that provided MSLQ translations for other languages (Jakesova & Hrbackova, 2014), we acknowledge the sample size might compromise the instrument’s reliability and validity. Thus, the interpretation of the findings should be considered in the context of this limitation.

Finally, our measurements were made during software usage, a context in which the understanding of student behavior without regulatory support still needs further investigation. This type of investigation will also be relevant to understand long-term performance.

## Conclusions

This study presented the design and evaluation of a computer-based learning environment for introductory programming education. The software aimed to support students by providing scaffolding grounded on the regulation of learning theory from an individual (self-regulation) and collective perspective (social regulation). We hypothesized that regulatory support can increase learning of computer programming and aid in development of regulation skills.

The results provide substantial evidence about the relevance of regulatory scaffolding for fostering learning. In addition, from a socio-cognitive perspective, the support for social regulation proved valuable for collaborative activities. However, no regulation skills development was observed, partially contradicting our initial hypothesis. Thus, as raised by previous literature, the mechanisms necessary for developing SRL skills during programming practice are still unclear.

Future research is necessary to investigate whether the effects maintain in the long run and without RESPE usage. In addition, there is also a need to understand the individual contributions of each scaffold in isolation from the others, an element little discussed in the current literature (Prather et al., 2020).

## Data Availability

The data that support the findings of this study are available from the corresponding author upon request.

## Footnotes

1 A common procedure to plan the program’s logic.

2 A conditional is a programming construct that controls the execution flow.

3 Test cases are formed by a set of inputs and expected outputs that students’ code should produce. This data is used to assess the exercise’s correctness.

4 The studies included in the analysis are listed in our Open Science Repository: https://osf.io/tu47x/?viewonly=aab2c35ee94d41b1abaf5ececea3f0dd.

5 https://www.acm.org/education/curricula-recommendations

6 The MSLQ translation to Portuguese is available in our Open Science Repository: https://osf.io/tu47x/?viewonly=aab2c35ee94d41b1abaf5ececea3f0dd.
